# Beyond silence: protocol for a randomized parallel-group trial comparing two approaches to workplace mental health education for healthcare employees

**DOI:** 10.1186/s12909-015-0363-9

**Published:** 2015-04-16

**Authors:** Sandra Moll, Scott Burton Patten, Heather Stuart, Bonnie Kirsh, Joy Christine MacDermid

**Affiliations:** 1 Institute for Applied Health Science, School of Rehabilitation Science, McMaster University, 4th Floor 1400 Main St.W., Hamilton, ON L8S 1C7 Canada; 2 Cumming School of Medicine, Department of Community Health Sciences, University of Calgary, Calgary, AB T2N 4Z6 Canada; 3 Public Health Sciences, Abramsky Hall, 3rd Floor, Queen’s University, Kingston, ON K7L 3 N6 Canada; 4 Department of Occupational Science and Occupational Therapy, University of Toronto, 500 University Ave, Unit 160, Toronto, ON M5G 1 V7 Canada; 5 School of Rehabilitation Science, McMaster University, Institute for Applied Health Science, 4th Floor, 1400 Main St. W, Hamilton, ON L8S 1C7 Canada

**Keywords:** Mental health, Workplace, Health promotion, Healthcare

## Abstract

**Background:**

Mental illness is a significant and growing problem in Canadian healthcare organizations, leading to tremendous personal, social and financial costs for individuals, their colleagues, their employers and their patients. Early and appropriate intervention is needed, but unfortunately, few workers get the help that they need in a timely way due to barriers related to poor mental health literacy, stigma, and inadequate access to mental health services. Workplace education and training is one promising approach to early identification and support for workers who are struggling. Little is known, however, about what approach is most effective, particularly in the context of healthcare work. The purpose of this study is to compare the impact of a customized, contact-based education approach with standard mental health literacy training on the mental health knowledge, stigmatized beliefs and help-seeking/help-outreach behaviors of healthcare employees.

**Methods/Design:**

A multi-centre, randomized, two-group parallel group trial design will be adopted. Two hundred healthcare employees will be randomly assigned to one of two educational interventions: Beyond Silence, a peer-led program customized to the healthcare workplace, and Mental Health First Aid, a standardized literacy based training program. Pre, post and 3-month follow-up surveys will track changes in knowledge (mental health literacy), attitudes towards mental illness, and help-seeking/help-outreach behavior. An intent-to-treat, repeated measures analysis will be conducted to compare changes in the two groups over time in terms of the primary outcome of behavior change. Linear regression modeling will be used to explore the extent to which knowledge, and attitudes predict behavior change. Qualitative interviews with participants and leaders will also be conducted to examine process and implementation of the programs.

**Discussion:**

This is one of the first experimental studies to compare outcomes of standard mental health literacy training to an intervention with an added anti-stigma component (using best-practices of contact-based education). Study findings will inform recommendations for designing workplace mental health education to promote early intervention for employees with mental health issues in the context of healthcare work.

**Trial registration:**

May 2014 - ClinicalTrials.gov: NCT02158871.

## Background

Mental illness in the workplace costs the Canadian economy an estimated $21 billion a year in reduced labour force participation [1]. It is associated with more lost work days than any other chronic condition, and the cost of mental health leave is, on average, double the cost of a leave for a physical illness [2]. Mental illness at work is a particularly costly issue for healthcare organizations. Healthcare workers in Ontario report high levels of workplace stress, and have a higher risk of mental health problems than any other occupational group [3,4]. Healthcare workers are more likely to miss work due to illness or disability and tend to be absent for significantly more days than workers in other sectors [5]. In addition, a high proportion of healthcare workers continue to work despite mental health problems [6]. Working despite mental illness, or presenteeism, can be more costly than absenteeism. Presenteeism can lead to poor work quality, interpersonal conflicts, and on the job errors and accidents [7]. Concerns about patient safety and quality of patient care have prompted calls for action to address this growing problem in healthcare organizations [8].

### Importance of early intervention for mental health issues

Despite the high prevalence and significant impact of mental health issues in healthcare work, the issues are often surrounded by secrecy and silence [9]. Many healthcare workers are reluctant to admit that they are ill, and do not seek help for their mental health problems when needed [6,10]. There is often a long lag time between the onset of symptoms and seeking treatment [11,12].

When a worker is struggling, managers and co-workers might not say or do anything because they do not recognize the signs of mental illness, they do not know how to respond, and/or they may judge the worker as “bad” rather than “ill” [13,14]. In healthcare, there can be a discourse of professional competence where it is not acceptable to admit the need for help [9,15]. Consequently, many workers do not get the help or support that they need and mental health issues can escalate to the point of crisis before they are addressed. Early intervention is critical to prevent the personal, social and financial costs of untreated mental health issues at work [16].

**Figure 1:**
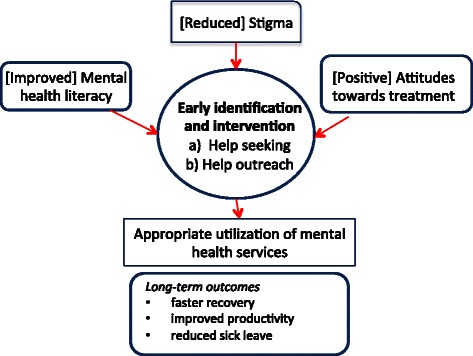
Conceptual Model of Early Intervention.

Early intervention, as defined in this study, is facilitated by knowledge, attitudes and behaviors that facilitate timely, effective and appropriate support for workers with emerging signs and symptoms of mental health problems or disorders [17]. Support may involve a range of options, from counseling and/or medication from a healthcare provider to connection with an Employee Assistance program or a self-help group. The conceptual model of early intervention guiding the project is outlined in Figure 1. Earlier intervention can be accomplished by augmenting two key health access behaviors; a) workers seeking help when they are struggling with their own mental health issues, and b) workers facilitating appropriate outreach to colleagues who are struggling in order to facilitate their help seeking behavior. Appropriate help seeking and outreach behaviors are considered together because they are the two primary mechanisms by which a worker receives the help that he/she needs, and they share common pathways [6]. Further, actions taken to facilitate early identification and intervention are mediated through reduction of stigma, improved mental health literacy and better attitudes towards professional treatment. This can have a dual effect on both help seeking and appropriate outreach to colleagues. Although the primary outcome of interest in this study is help seeking and outreach behavior, it should be noted that early intervention leads to more appropriate utilization of mental health services and faster recovery from mental health issues. The overall impact of greater efficiency, reduced absenteeism and sick leave, and increased productivity at work should provide economic advantages to both the worker and the employer [13]. Evaluation of these long-term outcomes is beyond the scope of this project, but will be the focus of subsequent research.

As outlined in the conceptual model, there are several key forces that may shape the nature and extent of early intervention for mental health problems. Mental health literacy, stigma, and beliefs about the value of seeking help are some of the key forces to consider [18]. Mental health literacy refers to the knowledge and skills required for recognition, management and/or prevention of mental disorders [19,20]. Literacy is a critical issue because many mental health problems are misunderstood or over-looked, therefore early identification is the first critical step in early intervention. Stigma, in the form of negative attitudes and stereotypical beliefs about mental illness is another key issue. Co-workers often judge workers with a mental illness and ostracize them rather than provide support [21]. These attitudes and beliefs contribute to fear of discrimination and are a substantial barrier to seeking help [22]. Another force that may shape behavior is one’s belief about the role and effectiveness of psychological treatment. If there is little faith in the value of a treatment approach, then a worker may not choose to pursue the treatment, and likewise a colleague may not recommend it to a co-worker who is struggling [18].

It should be noted that the model depicts a linear relationship between the key variables, however these relationships can be quite complex, and not always predictable. For example, mental health literacy training may or may not affect stigmatized beliefs; a decrease in stigma may not affect behavior; and experience in seeking help may reinforce or change beliefs about the value of a particular approach [23].

### Approaches to promote workplace mental health support

Many programs to promote early intervention for mental ill health at work are based on principles of mental health literacy training. Typically one or two sessions are scheduled to train stakeholders in the workplace about how to identify and respond to employees with mental health problems. Mental Health First Aid (MHFA) is one example of a well-established, standardized mental health literacy program that has been implemented and evaluated internationally in a variety of settings, including workplaces [24]. Outcomes of quasi-experimental and experimental studies of Mental Health First Aid training include improvement in recognition of mental disorders and their treatment, increased confidence in providing help for others, and increased helping behavior [25].

Although literacy initiatives show promise, they might not be sufficient in a healthcare environment. Many healthcare workers have reasonably high levels of mental health literacy. In fact, having a medical understanding of mental disorders appears to increase rather than decrease stigma and social distance, perhaps because the illness is perceived as fixed and chronic [26]. Stigma is reported to be quite high among healthcare workers [21]. Improvements in mental health literacy do not necessarily translate into reduced stigma or increased social inclusion [23]. In order to address the stigma associated with mental illness, contact-based education is recommended as a best practice approach [23]. Contact-based education (CBE) is a knowledge translation strategy that creates opportunities for positive interpersonal contact with someone who has personally experienced mental health issues [27]. Key ingredients for CBE include voluntary, positive, prolonged contact with a respected peer of equal status [28,29]. Positive interactions with a respected colleague who has personally experienced mental illness can disconfirm negative stereotypes, and the opportunities for active discussion can break down “us-them” barriers [28]. Evaluation studies of CBE with a range of student groups have reported positive outcomes, including a significant reduction in prejudice and social intolerance [27,30]. The impact on willingness to seek help, however, is not as clear [30].

Contact-based education led by a respected peer is a promising strategy to build literacy, promote positive attitudes, and reduce stigma, however, additional research is needed to systematically evaluate this approach and its impact on help-seeking/outreach behavior. A review of research on contact based education highlighted the potential of both retrospective and prospective contact, but also pointed to many methodological limitations in the literature and the need to examine the nature of contact [28]. There are no empirical evaluations of CBE in a workplace setting. One of the challenges is that CBE is more of a philosophy than a standardized approach to training. Many of the reports in the literature refer to single sessions involving personal story telling with a target audience, rather than the recommended principles of prolonged contact and active discussion [28]. This is a serious concern since definition and adherence to the active ingredients of intervention during its evaluation are fundamental to valid conclusions about its effectiveness. In this study, we propose to evaluate a workplace education program that integrates principles of CBE, including opportunities for ongoing active dialogue and discussion regarding the complex issues that shape the beliefs and behaviors of employees in healthcare organizations. According to principles of knowledge translation, a program is more likely to be effective if it is developed in partnership with stakeholders and tailored to the needs of the target audience [31]. In this study, we propose to evaluate a novel program called “Beyond Silence”, which was created to build on best practice principles of CBE as well as adult learning theory and knowledge translation in a healthcare setting. The plan is to compare its effectiveness to standard MHFA training that does not incorporate a contact-based education approach.

### Research questions

1. Is customized, contact-based education more effective than standard mental health literacy training in increasing the help-seeking/outreach behaviors of workers in a healthcare setting?
2. What mediates the impact of the contact-based intervention?
  - What is the effect of mental health literacy?
  - What is the effect of attitudes towards coworkers with mental health issues?
  - What is the effect of attitudes towards seeking professional treatment?
3. What process issues need to be considered in implementing mental health education in a healthcare workplace?

## Methods/Design

A multi-centre, randomized, evaluator-blinded, two-group parallel design will be adopted, comparing the impact of the Beyond Silence (CBE) program with mental health literacy training. A parallel group design will be adopted where participants in both groups will receive 12 hours of group-based education, but the content, schedule and format will be different. The 1:1 randomization sequence will be generated off site (in Calgary) using the software Stata. Randomization will occur in four blocks of 26 and four blocks of 24, leading to n = 100 in each assignment group and exactly 12 or 13 participants in each assignment. The sequence will not be shared with the investigative team, who will instead determine each participant’s assignment using an interactive web-based system. Mental Health First Aid training (MHFA) will serve as the control group since its efficacy is already well established in the literature [32]. The “Beyond Silence” approach is novel, and based on best practice principles, but research is needed to determine whether it is effective. Help seeking/outreach behavior, mental health literacy, and mental health stigma will be assessed at 3 month intervals: at baseline, after completion of the program and at 3 months follow-up. The study protocol was registered in May 2014 on ClinicalTrials.gov (ID#: NCT02158871). Ethical approval was also obtained through the Hamilton Integrated Research Ethics board.

### Participants

The study will be conducted in two healthcare organizations in the same mid-sized urban center in southern Ontario. One is a large healthcare facility that employs approximately 10,000 full and part-time workers across five sites. The other organization is a mid-sized hospital with approximately 4000 employees across three main sites. Volunteer participants will be recruited through posters, staff newsletters, intranet sites, information sessions with program managers, and email follow-up. Inclusion criteria include: a) full, part-time or casual employment in any area of the organization, b) agreeable to being randomly assigned to either of the two programs, c) commitment to attending the 12 hours of mental health education outside of paid work time, and d) no prior training in either program. Screening will be completed by the project coordinator either via email or by telephone. Recruitment and random assignment to the two groups will continue over four intake periods in each organization, according the sample justification outlined below. Consent will be obtained from each participant prior to completion of the each online survey (pre, post and at 3-month follow-up), with a hard copy of the consent form provided at the start of the first in-person session.

The plan is to enroll 200 employees in total, with 100 participants in each group. In each of the four intake periods, we plan to include 24 to 26 participants, and will actively recruit to ensure sufficient numbers prior to randomization. Following randomization, an intent-to-treat analysis will be incorporated, including all study participants. Quantitative precision estimates suggest that this sample size is sufficient to detect any real and important benefit of CBE over literacy training on change in help-seeking behavior. Two RCT evaluations of MHFA (literacy) interventions among Australian healthcare workers found that 50% to 75% of workers reportedly offered help to a person with mental health problems, prior to the intervention [24,25]. Based on this, we expect an average of about 1–2 behaviors at baseline across the samples. To simplify, we approximate the precision to detect change in mean count from baseline to the 6 month follow-up using calculations for the linear mixed-effects model, but allowing for non-constant variance of the counts by assuming that the variance averaged over groups and occasions might be as high as 4. Also assuming high consistency of help-seeking within groups (test-retest correlation, 0.80), we have an 80% chance of finding a significant (p < .05) difference between the two groups in average change over 6 months of as little as 0.44 behaviors. That is, even if the real advantage of CBE over MHFA is very small (less than 1 new behavior, on average), we have a high probability of detecting it. The precision for estimating mediators such as changes in stigma scores is similarly high. Based on initial pilot data, there were significant increases in behavior over time therefore the projected sample size should be more than sufficient to detect change.

Compliance with the programs will be tracked through attendance rates, with 75% attendance considered to be good compliance. To minimize withdrawals or missing data, multiple strategies will be put in place to allow participants to respond (written, electronic) with incentives for survey completion and proactive follow-up by email and/or phone. Based on the level of engagement of pilot participants, minimal loss to follow-up is expected, but we will impute assuming random missing-ness if needed. The repeated measures mixed effects model accounts for missing data under a missing at random assumption, such that participants with a single missing follow-up survey do not need to be excluded from the data analysis.

### Interventions

The ***“Beyond Silence” CBE intervention*** program will be co-led by trained peer educators who have personally experienced mental ill health and recovery. The peer educators will be employees within the organization who not only have personal experience with mental ill health (either themselves or a close family member), but are good communicators and credible leaders within the organization. They will be recruited and trained to effectively teach the content, share their personal experiences, and facilitate discussion. Four employees (plus one “back-up” person) will contracted for approximately 125 hours over the course of the two years (including training and support sessions), with two peer educators facilitating each Beyond Silence group program. The Beyond Silence curriculum was designed to address the unique needs of the organization and is based on; a) pilot data from the initial qualitative phase of the project [33], b) a review of programs utilized in other workplaces, and c) best practice principles of contact based education and knowledge translation. It will include a series of 6 in-person group sessions (plus 5 online sessions) designed to provide information, diminish anxiety and promote empathy; all best-practice dimensions of CBE [28]. Sessions will be held weekly, alternating between a 1.5-2 hour in-person session and an online learning opportunity. The in-person sessions will combine information sharing and discussion, using workplace-based vignettes to build mental health literacy, reduce stigma and increase confidence and skill in reaching out to seek/offer help. The online “virtual” sessions using a secure, online platform, will encourage self-directed exploration of community and online resources and provide an opportunity for online dialogue that can be accessed at any time or place. All participants will receive a program handbook with the core content and worksheets. Each Beyond Silence program will be offered over a three month period. Over the course of the project, eight programs will be offered with approximately 12–13 participants per program.

The control group intervention is Mental Health First Aid (MHFA), a standardized, twelve-hour educational program designed to teach participants how to recognize the early warning signs of mental illness, how to provide initial help to someone in a mental health crisis, and how to support people who are developing mental health problems [25]. It is an evidence-based approach, with several RCT’s documenting significant increases in mental health literacy, decreased social distance, and increased reports of helping behaviors [24,34,35]. The program originated in Australia, but is being implemented across Canada, under the leadership of the Mental Health Commission of Canada. A staff member in each organization who has been trained through the 5-day instructor-training program will be seconded to facilitate the program within their organization. Each course will be offered as two full days, typically one week apart. All participants will receive a training manual, which covers all of the content of the standardized, module-based curriculum. Over the course of the project, eight courses will be offered with approximately 12–13 participants per course.

The location and specific dates/times for the sessions will be customized for each group in order to facilitate participation and maximize response rate, but groups will scheduled to start in regular intervals over the course of the two years (fall, winter, spring, fall). The Beyond Silence programs will be scheduled in the early evenings (outside of regular work hours), and the MHFA programs will be scheduled for two full days, one week apart.

Monthly meetings will be scheduled with the peer educators to provide support, address any questions/concerns, and maximize fidelity to the principles of the Beyond Silence program. A fidelity measure was designed to track adherence to structural elements (6 items), as well as content and process principles (10 items) of the program, using a 4-point Likert-type rating scale. Fidelity assessment will occur between sessions 3 and 5, and will be completed by a participant observer who is part of the core project team. Ratings will be discussed with peer educators at monthly meetings, with any departures from the established principles noted and explained, including plans (if applicable) to improve adherence. Fidelity to the MHFA program principles will be monitored through MHFA Canada, as per their internal processes.

### Data collection

Each participant will complete a survey prior to starting the training, immediately following the intervention, and three months following completion of the program. Participants will have the option of completing a written or online version of the survey that is sent directly to them by email and/or letter. Proactive outreach, with two follow-up reminders and a gift card incentive will be used to maximize return rates. Baseline data for the MHFA training will take place three months prior to initiation of the two-day program, so that the post-test measurements in the MHFA and Beyond Silence groups occur concurrently.

The survey will include 6 main sections. Content and data collection methods will be identical in the two groups:

- **Demographic data** (gender, age, education, ethnicity, job tenure, position) will be gathered to obtain a basic profile of participants.
- **Mental health experience** – Participants will be asked whether they have a history of mental health issues, either in themselves or a family member (yes/no), the nature of these issues (examples provided), and any personal experiences with mental health issues over the past six months (yes/no). The presence of mental health issues is based on self report, and will be defined as changes in thinking, mood or behavior that impair day-to-day functioning [34]. This lay definition and examples will be provided to capture a range of experiences that may or may not have been formally diagnosed by a medical practitioner.
- **Help-seeking behavior** – Participants will be asked to report whether they accessed any services from a list of 10 health, workplace and community service options. Questions regarding service utilization are adapted from the 2012/2012 Canadian Community Health survey [36], in order to provide a population reference, although several work-related services (e.g. EAP, union) are added as an option. Participants may endorse more than one of the behaviors, and a summative score of the number of behaviors will be used to measure change in help-seeking. Since the incidence of reported help seeking may be low over the time frame of the study, attitudes or intent to seek help will also be tracked. Intention to seek psychiatric help is a significant predictor of behavior [18]. The Attitudes Toward Seeking Professional Psychological Help Scale–short form (ATSPPHS), a 10 question survey using a four point Likert-type scale response will be used to track beliefs and intent to seek professional help [37]. It is a widely used scale with good internal consistency (α = .78), and criterion validity supported by links between scale scores and mental healthcare use [37].
- **Outreach behavior** – Baseline and follow-up data will be gathered regarding personal contact with a co-worker about mental health problems (yes/no), and any contact over the past 6 months (yes/no). If contact did occur, participants will be asked whether they provided help to the co-worker, and if so, to identify the type of help from a list of 10 possible options. The list of “outreach” behaviors was based on the ones used for evaluation of the MHFA program (e.g. ‘spent time listening to problem’, ‘recommend professional help’) [34], with an adaptation to include a several work-specific options (e.g., ‘recommend EAP’, ‘offered assistance with job duties’). Participants are invited to check all behaviors that apply (with an open-ended option), and scoring is based on one point per action, with a summative score used to track change. In addition to the behavior list, participants will be asked to rate their confidence in providing help on a 7-point Likert scale.
- **Stigma towards co-workers with mental illness** – The MHCC Opening Minds Scale for Healthcare Providers will be used; a 20-item questionnaire that was designed to evaluate the attitudes of healthcare providers towards people with mental illness [38]. The tool has good internal consistency (α = .82), and satisfactory test-retest reliability (ICC = .66), with limited impact of social desirability [38]. This tool is being implemented in sites across the country by researchers with the Mental Health Commission Opening Minds team; therefore data from this project can be linked with the larger database.
- **Mental Health literacy** – Four vignettes of employees with workplace mental health issues will be used to assess awareness of issues and when/how to respond to these issues in the workplace. The vignettes are adapted for a healthcare workplace based on a review of vignettes reported in the literature study [33,39,40], as well as analysis of key issues reported in the pilot phase of the study. Vignettes will incorporate differences in gender, nature and severity of illness, and a 7-point Likert scale from novice to expert will be used to track key dimensions of literacy, including perceived knowledge about the condition, what to say/do and what resources to access [33].

In addition to quantitative outcome data, qualitative data will be collected to track program implementation. Attendance will be recorded at each session, in order to understand patterns of participation and potential dose–response impact. At the end of each group, all participants will be asked to provide written feedback (as part of the post-group survey) regarding their experience, including perceived strengths of the program and suggestions for improvement. Feedback will also be gathered from peer educators regarding the program and their experience as facilitators. In the monthly support meetings, the project coordinator will inquire about and document notes regarding issues raised by the peer educators. As outlined earlier, monthly supervision meetings will also be used as a way of tracking fidelity to the program principles.

### Data analysis

In accordance with Research Question 1, we hypothesize that help-seeking behavior will increase from baseline to 3 and 6 months by a greater amount in the Beyond Silence group than in the MHFA control. The primary approach to this question is the repeated measures analysis of a checklist of desirable help seeking/help providing behaviors, obtained from the survey at each time point. Overall tests of the change across all three measurements are available from generalized linear mixed-effects analysis, assuming that the count (from 0 to 20 behaviors) is distributed as a Poisson variable. This is a standard approach that accounts for over-dispersion in the Poisson model arising from the repeated measurements, permits the inclusion of participants who have not completed all three assessments, and allows for flexible modeling of the change over time and the inclusion of any relevant covariates [41]. In the primary analysis, the statistical significance of treatment group in this model will be used to address the first research question. In view of the randomized design, the primary analysis will not include additional covariates, but the model will be used to explore modifying effects by including additional covariates (e.g. age, sex, prior experience with mental illness) and interactions between these covariates and treatment group. As a secondary outcome, we will analyze a scaled measure of behavioral intentions using similar linear mixed-effects analysis.

In Research Question 2, we propose mediating forces that are expected to be different in the two programs, including scaled measures of mental health literacy (expected to be higher in the MHFA group), stigma regarding mental illness (expected to be greater in the Beyond Silence group), and attitudes towards seeking professional help (expected to be higher in the Beyond Silence group). Following recommendations by Baron and Kenny [42], we will initially explore whether changes in literacy occur in association with the interventions. These analyses will use linear regression, modeling change in literacy and stigma, respectively, occurring in association with the interventions. Next, we will use Poisson or negative binomial regression to determine whether changes in literacy, stigma and attitudes associated with intervention (as well as overall levels of each) predict counts on the desirable behavior checklist. If associations are observed in both cases, we will evaluate change in these measures using linear mixed effects as described for Research Question 1. Substantial diminishment, or disappearance, of the effect of the interventions on help-seeking when the potential mediators are included in the model will be interpreted as providing evidence of mediation.

The random assignment of participants to intervention groups eliminates biases in the evaluation of the treatment effects, but the generalizability of this effect in relation to the representativeness of the sample will be assessed by comparing demographic and employment data from study participants to the equivalent data available for the entire organization. This data also provides insight on the reach of the program within the organization in terms of the characteristics of who volunteers to participate in this kind of program. This augments the qualitative analysis of Research Question 3.

Although our primary research questions are analyzed through a clinical trial, a mixed methods approach can be particularly important for understanding program implementation. Research Question 3 addresses the process of implementation; input from program participants and leaders will add depth to our understanding of the mediators and effectiveness of implementation. First, written feedback from the post-group surveys for each intervention will be reviewed and coded to identify common themes regarding program impact, strengths and areas for improvement. Next, notes from monthly meetings with the peer educators will be reviewed to identify implementation strengths and challenges from the perspective of the facilitators [43]. Key themes from each of the programs will be compared and contrasted to understand the differential impact and critical ingredients of MHFA versus contact-based education. The qualitative data (including attendance records) could be used to corroborate (or refute) quantitative findings about the differential impact of each approach.

## Discussion

Overall, study findings will be used to analyze the relative value of the Beyond Silence program in promoting early intervention for healthcare workers who are struggling with mental health issues. Future research will explore larger scale implementation in other workplaces, and the longitudinal impact on organizational indicators of productivity.

It is hypothesized that healthcare workers have higher levels of mental health literacy than the general public, therefore contact-based education will be more effective than literacy training in addressing the complex forces that prevent individuals from seeking help for their own mental health problems, or provide help to others who are struggling. Most current studies track knowledge and attitude change, therefore the focus of this study on behavior change (help-seeking and help-outreach) and the forces that contribute to behavior change will add depth to our current understanding of the impact of employee educational intervention.

## References

[1] Conference Board of Canada. Mental health issues in the labour force: Reducing the economic impact on Canada. 2012. http://www.conferenceboard.ca/e-library/abstract.aspx?DID=4957. Accessed Feb 2014.

[2] Dewa CS, Chau N, Dermer S (2010). Examining the comparative incidence and costs of physical and mental health-related disabilities in an employed population. J Occup Environ Med.

[3] Statistics Canada (2006). 2005 national survey of the work and health of nurses (CIHI share file).

[4] Wieclaw J, Agerbo E, Mortensen PB, Bonde JP (2006). Risk of affective and stress related disorders among employees in human service professions. Occup Environ Med.

[5] Canadian Institute for Health Information (CIHI) (2005). Canada’s healthcare providers: 2005 Chartbook.

[6] Gärtner FR, Nieuwenhuijsen K, van Dijk FJ, Sluiter JK (2010). The impact of common mental disorders on the work functioning of nurses and allied health professionals: a systematic review. Int J Nurs Stud.

[7] Attridge M (2008). A quiet crisis: the business case for managing employee mental health.

[8] Silas L (2007). From promise to practice: getting healthy work environments in health workplaces. Healthcare Papers.

[9] Moll S (2010). Mental health issues and work: Institutional practices of silence in a mental healthcare organization. Doctoral thesis.

[10] Crout LA, Chang E, Cioffi J (2005). Why do registered nurses work when ill?. J Nurs Adm.

[11] Kessler RC, Frank RG (1997). The impact of psychiatric disorder on work loss days. Psychol Med.

[12] Lesage A, Dewa CS, Kirsh B (2006). The momentum for research on mental health in the workplace in Canada. Can J Comm Mental Health.

[13] Bender A, Kennedy S (2004). Mental health and mental illness in the workplace: diagnostic and treatment issues. Healthcare Papers.

[14] Krupa T, Kirsh B, Cockburn L, Gewurtz R (2009). Understanding the stigma of mental illness in employment. Work.

[15] Rosvold EO, Bjertness E (2001). Physicians who do not take sick leave: hazardous heroes?. Scand J Publ Health.

[16] Pomaki G, Franche R-L, Murray E, Khushrushahi N, Lampinen TM (2012). Workplace-based work disability prevention interventions for workers with common mental health conditions: a review of the literature. J Occup Rehab.

[17] Commonwealth Department of Health and Aged Care. Promotion, Prevention and Early Intervention for Mental Health—A Monograph. Mental Health and Special Programs Branch, Commonwealth Department of Health and Aged Care, Canberra; 2000. http://findahealthservice.act.gov.au/c/fahs?a=dlpubpoldoc&document=823. Accessed Jan 2014.

[18] Schomerus G, Matschinger H (2009). Angermeyer MC attitudes that determine willingness to seek psychiatric help for depression: a representative population survey in applying the theory of planned behavior. Psychol Med.

[19] Jorm AF, Korten AE, Jacomb PA, Christensen H, Rodgers B, Pollitt P (1997). “Mental health literacy”: a survey of the public’s ability to recognize mental disorders and their beliefs about the effectiveness of treatment. Med J Aust.

[20] Kelly CM, Jorm AF, Wright A (2007). Improving mental health literacy as a strategy to facilitate early intervention for mental disorders. Med J Aust.

[21] Joyce T, Hazelton M, McMillan M (2007). Nurses with mental illness: their workplace experiences. Int J Mental Health Nurs.

[22] Schomerus G, Angermeyer MC (2008). Stigma and its impact on help-seeking for mental disorders: what do we know?. Epidemiol Psichiatr Soc.

[23] Stuart H, Arboleda-Florez J, Sartorius N (2012). Paradigms lost: fighting stigma and the lessons learned.

[24] Kitchener B, Jorm AF (2004). Mental health first aid training in a workplace setting: a randomized controlled trial. BMC Psychiat.

[25] Kitchener B, Jorm AF (2002). Mental health first aid training for the public: evaluation of effects on knowledge, attitudes and helping behavior. BMC Psychiat.

[26] Hugo M (2001). Mental health professionals’ attitudes towards people who have experienced a mental health disorder. J Psychiatr Ment Health Nurs.

[27] Corrigan PW, River L, Lundin RK, Penn DL, Uphoff-Wasowski K, Campion J (2001). Three strategies for changing attributions about severe mental illness. Schizophr Bull.

[28] Couture SM, Penn DL (2003). Interpersonal contact and the stigma of mental illness: a review of the literature. J Ment Health.

[29] Wallach HS (2004). Changes in attitudes towards mental illness following exposure. Comm Ment Health J.

[30] Stuart H, Koller M, Christie R, Pietrus M (2011). Reducing mental health stigma: a case study. Healthcare Q.

[31] Graham ID, Logan J, Harrison MB, Straus SE, Tetroe J, Caswell W (2006). Lost in knowledge translation: time for a map?. J Cont Ed Health Prof.

[32] Kitchener B, Jorm AF (2006). Mental health first aid training: review of evaluation studies. Aust N Z J Psychiat.

[33] Moll S (2014). The web of silence: a qualitative case study of early intervention and support for healthcare workers with mental ill-health. BMC Public Health.

[34] Jorm AF, Kitchener BA, Fischer J-A, Cvetkovski S (2010). Mental health first aid training by e-learning: a randomized controlled trial. Aust N Z J Psychiat.

[35] Jorm AF, Kitchener BA, O’Kearney R, Dear KBG (2004). Mental health first aid training of the public in a rural area: a cluster randomized trial. BMC Psychiat.

[36] Statistics Canada. The 2011/2012 Canadian Community Health Survey. 2012. http://www.statcan.gc.ca/daily-quotidien/131113/dq131113c-eng.htm. Accessed Aug 2013.

[37] Elhai JD, Schweinle W, Anderson SM (2008). Reliability and validity of the attitudes toward seeking professional psychological help scale-short form. Psychiatry Res.

[38] Kassam A, Papish A, Modgill G, Patten S (2012). The development and psychometric properties of a new scale to measure mental illness related stigma by health care providers: the opening minds scale for health care providers (OMS-HC). BMC Psychiat.

[39] Schomerus G, Matschinger H, Angermeyer MC (2013). Continuum beliefs and stigmatizing attitudes towards persons with schizophrenia, depression and alcohol dependence. Psychiatry Res.

[40] Swami V (2012). Mental health literacy of depression: gender differences and attitudinal antecedents in a representative British sample. PLoS One.

[41] Diggle PJ, Heagerty P, Liang K, Zeger SL (2002). Analysis of Longitudinal Data.

[42] Baron RM, Kenny DA (1986). The moderator-mediator variable distinction in social psychological research: conceptual, strategic and statistical considerations. J Pers Soc Psychol.

[43] Thorne S, Kirkham S, O’Flynn Magee K (2004). The analytic challenge in interpretive description. Int J Qual Methods.

